# RNF4~RGMb~BMP6 axis required for osteogenic differentiation and cancer cell survival

**DOI:** 10.1038/s41419-022-05262-1

**Published:** 2022-09-24

**Authors:** Rostislav Novak, Yamen Abu Ahmad, Michael Timaner, Eliya Bitman-Lotan, Avital Oknin-Vaisman, Roi Horwitz, Oliver Hartmann, Michaela Reissland, Viktoria Buck, Mathias Rosenfeldt, David Nikomarov, Markus Elmar Diefenbacher, Yuval Shaked, Amir Orian

**Affiliations:** 1 Rappaport Research Institute and Faculty of Medicine, Technion Integrative Cancer Center Technion- IIT, Haifa, 3109 610 Israel; 2 Rambam Health Campus Center, Haifa, 3109610 Israel; 3 Department of Pathology, University of Würzburg, Würzburg, Germany; 4 Protein Stability and Cancer Group, University of Würzburg, Department of Biochemistry and Molecular Biology, Würzburg, Germany

**Keywords:** Differentiation, Bone cancer

## Abstract

Molecular understanding of osteogenic differentiation (OD) of human bone marrow-derived mesenchymal stem cells (hBMSCs) is important for regenerative medicine and has direct implications for cancer. We report that the RNF4 ubiquitin ligase is essential for OD of hBMSCs, and that RNF4-deficient hBMSCs remain as stalled progenitors. Remarkably, incubation of RNF4-deficient hBMSCs in conditioned media of differentiating hBMSCs restored OD. Transcriptional analysis of RNF4-dependent gene signatures identified two secreted factors that act downstream of RNF4 promoting OD: (1) BMP6 and (2) the BMP6 co-receptor, RGMb (Dragon). Indeed, knockdown of either RGMb or BMP6 in hBMSCs halted OD, while only the combined co-addition of purified RGMb and BMP6 proteins to RNF4-deficient hBMSCs fully restored OD. Moreover, we found that the RNF4-RGMb-BMP6 axis is essential for survival and tumorigenicity of osteosarcoma and therapy-resistant melanoma cells. Importantly, patient-derived sarcomas such as osteosarcoma, Ewing sarcoma, liposarcomas, and leiomyosarcomas exhibit high levels of RNF4 and BMP6, which are associated with reduced patient survival. Overall, we discovered that the RNF4~BMP6~RGMb axis is required for both OD and tumorigenesis.

## Introduction

Regulation of cellular differentiation by the ubiquitin pathway is a fundamental process that is highly crucial to tissue regeneration and cancer [1, 2]. Enzymes within the pathway inhibit differentiation by mediating the degradation of key differentiation regulators. For example, the muscle-specifying transcription factor MyoD is degraded by the ubiquitin pathway, and its ubiquitination by the E3 ligase HUWE1 and subsequent proteasomal degradation inhibits muscle cell differentiation [3, 4]. Several ubiquitin ligases attenuate or inhibit osteogenic bone differentiation (OD), including Wwp1, Skp2, Smurf1, and Smurf 2 ubiquitin ligases. The latter target the key osteogenic transcription factor RUNX2 for degradation, resulting in inhibition of OD [5–11]. The involvement of deubiquitinating enzymes (DUBs, USPs) in osteogenic differentiation was also demonstrated; USP1 is a ubiquitin-specific protease that removes ubiquitin chains assembled on the Inhibitor of differentiation proteins, termed Ids, preventing their ubiquitin-dependent degradation and sustaining muscle progenitor stemness [12]. Moreover, in cancer, USP1 potentiates tumorigenesis of osteosarcoma cancer-initiating stem cells [12]. USP34 was, likewise, shown to regulate BMP2 signaling and inhibit OD [13]. While USP53, in contrast, promotes osteogenesis by inhibiting the degradation of β-catenin [14]. Less is known, however, regarding ubiquitin-ligase enzymes (E3) that actively promote OD.

We identified RNF4 as an essential E3 required for OD of human bone marrow-derived mesenchymal stem cells (hBMSCs). hBMSCs are a heterogenous population of somatic stem cells that can differentiate and give rise to bone, cartilage, adipose, fat, and muscle cells. hBMSCs can be isolated from bone marrow and undergo several stages of maturation, resulting in differentiated osteoblasts [15**–**17]. The process of OD is intrinsically regulated by factors within stem cells, as well as by factors secreted from the differentiating cells and the bone marrow microenvironment [18]. Among the prominent of these factors are the bone morphogenic/transforming growth factor β (BMP/TGFβ) protein superfamily. Mutation in these ligands or their receptors results in bone mal-development [19]. Of specific interest is BMP6, a master regulator of osteogenesis that induces the activation of the downstream SMAD pathway, promoting the expression of key regulatory transcription factors such as Cbfa1/Runx2 and Osterix, as well as that of osteoblast-specific proteins such as Type I collagen, osteocalcin, and bone sialoprotein. BMPs also activate osteogenesis via non-canonical MAPK signaling [19**–**21]. During OD, the differentiating hBMSCs increase the expression of RUNX2 and β-catenin, accumulate calcium precipitates, and increase alkaline phosphatase activity (ALP) [22].

Here we report that the ubiquitin ligase RNF4 is an essential regulator of OD. RNF4 is a SUMO-targeted ubiquitin ligase (STUbL) that has SUMO-dependent and independent interfaces/interactions with its substrates [23**–**26]. Within the N-terminal region of RNF4 are four SUMO-interacting motives that enable binding to polySUMO chains, thus, connecting SUMOylation with ubiquitination. An arginine-rich motif (ARM), located downstream to the SIM motifs, binds to phosphorylated proteins. The C-terminal region of the RNF4 contains a RING domain that catalyzes ubiquitination and a nucleosome-targeting region (NTR) that enables binding to nucleosomes [27]. RNF4 targets SUMOylated proteins for degradation [23, 27, 28] but also binds to phosphorylated oncoproteins via its ARM domain independent of de-novo SUMOylation. RNF4-dependent ubiquitination enhances oncoprotein stability and activity by catalyzing the formation of heterotypic ubiquitin chains [29]. In cancer, RNF4 has context-dependent tumor-suppressing or tumor-promoting functions. In acute promyelocytic leukemia, it is required for the degradation of the fusion oncoprotein PML-RARα, resulting in the differentiation of the leukemic cells [30, 31]. In contrast, RNF4 promotes both in vitro and in vivo tumorigenesis of epithelial cancers and confers resistance to receptor tyrosine kinase inhibitors (RTKi) in melanoma [32]. Breast cancer and melanoma patients exhibiting high levels of RNF4 proteins have poorer prognoses and exhibit resistance to combined RTKi therapy [29, 32]. While RNF4 is part of a stemness signature shared by stem cells [33], its role in stem cell differentiation, in sarcomas and mesenchymal tumors is unknown.

Here we discovered a critical role for the RNF4~RGMb~BMP6 axis in the OD of hBMSCs and tumorigenesis. RNF4 is required for the expression of BMP6 and its co-receptor RGMb (Dragon) mRNAs [34]. Remarkably, both proteins together play a critical role in hBMSC OD downstream of RNF4. Moreover, the RNF4~BMP6~RGMBP axis is critical for the survival of aggressive bone cancers and melanoma and is associated with poor prognosis of sarcoma patients.

## Results

### RNF4 is essential for hBMSC differentiation

**Fig. 1:**
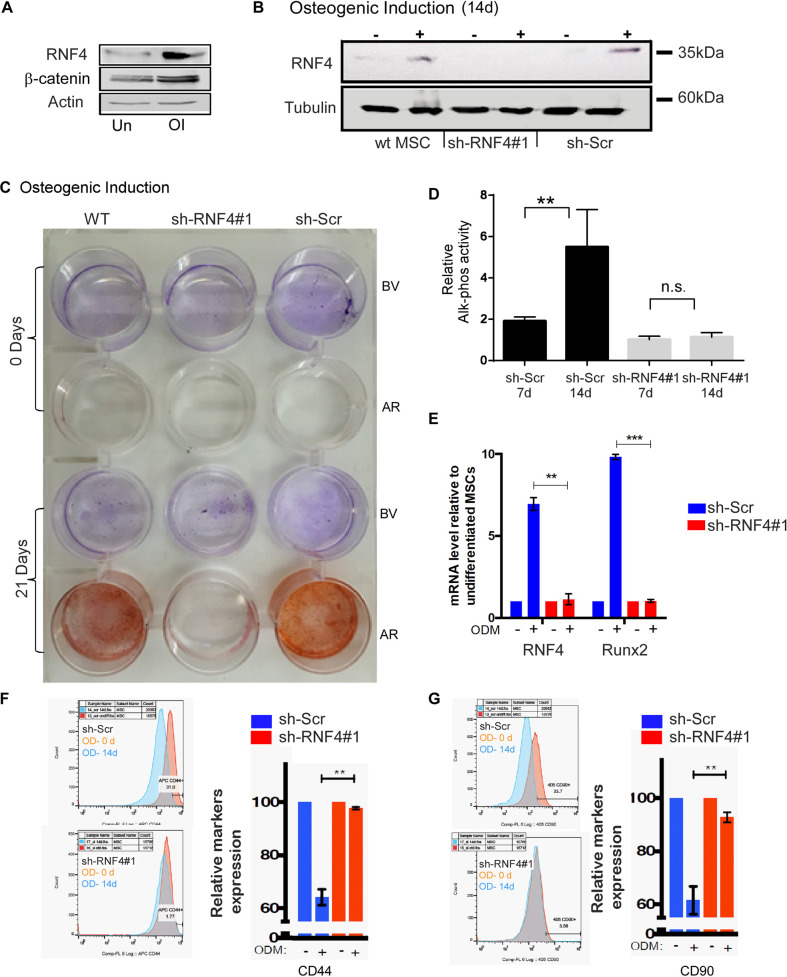
RNF4 is essential for OD of hBMSCs. **A** Western blot analysis of endogenous RNF4 and β−catenin levels in protein extracts derived from untreated hBMSCs (Un) or cells in which OD was induced (OI) for fourteen days; **B** Western blot analysis of endogenous RNF4 protein levels in protein extract derived from wild-type hBMSC or hBMSCs in which RNF4 was knocked down using shRNA (sh-RNF4#1) or control scrambled shRNA (sh-Scr) with or without OI for 14 days. Actin (A) and tubulin (B) serve as a loading control. **C** Accumulation of calcium precipitates during OD at day zero and 21 days of OI, as evident by Alizarin Red staining (AR, red) in control (sh-Scr) or hBMSCs in which RNF4 was knocked down using sh-RNA (sh-RNF4). Brilliant-Violate staining (BV, purple) marks viable cells. **D** Alkaline phosphate activity upon 7, 14 days of OI of control scrambled treated hBMSCs, or hBMSCs in which RNF4 was knocked down using shRNA (*n* = 2 **p < 0.01; ns non-significance). **E** qPCR analysis of RNF4 and RUNX2 mRNA levels in control (sh-SCR) or RNF4-targeted hBMSCs (sh-RNF4). **F**, **G** FACS-assisted analysis of OD of the indicated cell surface markers at day zero and 14 days of OD. In both **F** and **G** a representative FACS analysis is shown, and Bar-graph represents three independent biological experiments. CD44 and CD90 are markers of undifferentiated hBMSCs, *n* = 3 ***p < 0.001; **p < 0.01.

To investigate a potential role for RNF4 in OD, we induced the differentiation of hBMSCs towards the osteogenic lineage and osteoblast formation and monitored the expression of RNF4, as well as the expression of the osteogenic transcription factor RUNX2 and β-catenin. hBMSC populations were grown in control or differentiation media, and protein levels were monitored at indicated times. Low levels of RNF4 and β-catenin proteins were observed in progenitors, increasing upon osteogenic differentiation (Fig. 1A). The mRNA level of RUNX2 also increased during OD (Fig. 1E).

To examine whether RNF4 is required for hBMSC OD, we knocked down RNF4 in hBMSCs using two independent anti-RNF4 shRNAs (shRNF4#1, #2) or a scrambled control that we used previously (Fig. 1B and Supp. Fig. 1C). During OD, hBMSCs were examined for calcium hydroxyapatite aggregate levels, visualized using Alizarin Red (AR), and for the activity of the bone-related enzyme alkaline phosphatase (ALP), as biological markers for OD [21]. Upon OD, wild-type and control (scrambled shRNA) cells accumulated calcium precipitates (Fig. 1C) and exhibited increased ALP activity (Fig. 1D). In contrast, hBMSCs in which RNF4 was eliminated using shRNF4 were only minimally positive for AR and did not exhibit an increase in ALP activity (Fig. 1C, D). Likewise, the mRNA level of the osteogenic transcription factor Runx2, which increased upon OD in control differentiating cells, remained low in RNF4-deficient hBMSCs (Fig. 1E). We also noticed that while the initial cell density was identical at seeding, the proliferation of RNF4-deficient hBMSCs was only minimally lower compare with naive cells during OD, suggesting that they did not enter the proliferative expansion stage that is associated with hBMSC differentiation as observed during mesenchymal differentiation [35].

To further characterize the cell identity of RNF4-deficient hBMSCs, we determined the protein levels of CD44 and CD90, which are established surface markers of undifferentiated hBMSCs and found that the expression of these receptors declines upon OD [36, 37]. Flow-cytometry analysis established that upon fourteen days of OD, CD44 and CD90 levels declined in control hBMSC cells but did not decline in RNF4-deficient hBMSCs (Fig. 1F, G). Moreover, we observed only minimal expression of CD31 and CD45, that are non-hBMCs hematological markers and their expression was not affected by the elimination of RNF4 in hBMCs (Supp. Fig. 1D–F). We concluded that hBMSCs lacking RNF4 fail to differentiate and are stalled as undifferentiated progenitors.

### Secreted RNF4-dependent factors are required for OD

**Fig. 2:**
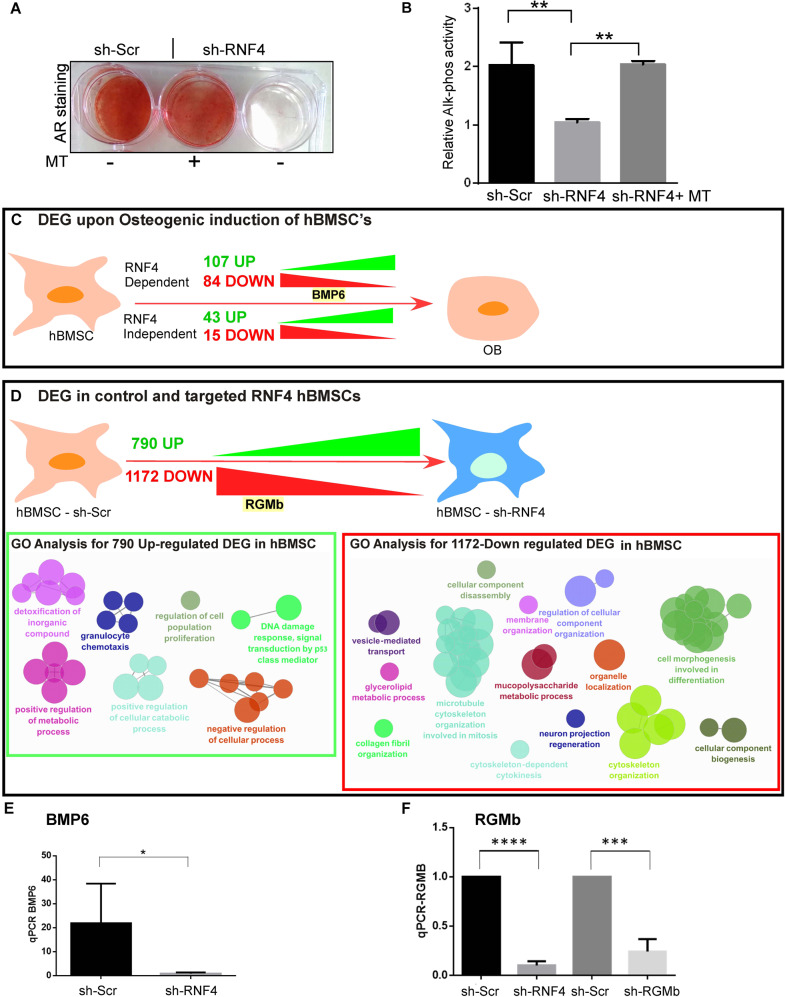
RNF4-dependent secreted factors are required for hBMSC differentiation. **A** Media transfer restores differentiation of RNF4-deficient stalled progenitors. OD assisted by transfer of culture media from the indicated cells for 21 days of the indicated hBMSCs, as analyzed by AR staining. MT: transfer of culture media collected from wild-type hBMSCs (MT−) or hBMSCs undergoing OD (MT+) (see Methods for details). sh-Scr denotes scrambled control shRNA; sh-RNF4 denotes sh-RNF4. **B** Alkaline phosphatase (ALP) activity of the indicated hBMSCs under experimental conditions similar to **A**. **C**, **D** Identification of RNF4-regulated genes during OD and in hBMCs. **C** Schematic diagram of RNA-seq results of OD in control hBMSCs, or RNF4-deficient hBMSCs; OB osteoblasts. **D** Schematic diagram of RNA-seq results of control hBMSCs or RNF4-deficient hBMSCs, lower panels depict key GO groups of RNF4-regulated genes in hBMSCs. **E**, **F** qPCR analysis of BMP6 (**E**) and RGMb (**F**) mRNA levels in the indicated hBMSCs. BMP6 levels were determined 2 weeks after OI and RGMb levels in undifferentiated progenitors. sh-SCR; scrambled shRNA, control) *n* = 3 ****p < 0.0001; ***p < 0.001; *p < 0.1. Statistics was calculated by one-step ANOVA Graph-prism 6.

RNF4 activity is tightly related to its ability to enhance gene expression, acting predominantly as a co-activator as well as enabling de-repression [29, 32, 38, 39]. We hypothesized that RNF4 promotes OD by enhancing the expression of secreted proteins from differentiating hBMSC that act locally. To test this hypothesis, we examined the ability of conditioned media, collected from wild-type hBMSCs that underwent OD for 21 days, to alleviate the differentiation block imposed on hBMSCs in which RNF4 was knocked down by shRNF4. As seen in Fig. 2A, B, while RNF4-deficient hBMSCs failed to differentiate, incubation of these cells in filtered culture media from wild-type differentiating hBMSCs (MT+), but not in media from undifferentiated progenitors (MT−), restored OD, as determined by increased AR staining and increased ALP activity (Fig. 2A, B). Thus, it is likely that the conditioned media from wild-type differentiating hBMSCs contained RNF4-induced factors that enabled full OD of these stalled RNF4-deficient hBMSCs.

We then performed RNA-seq analysis to determine a potential transcriptional role for RNF4 in OD and to identify RNF4-regulated secreted factors. We compared the changes in the gene signature of scrambled Scr-control hBMSCs with that of hBMSCs in which RNF4 was knocked down using shRNF4#1 upon OD.

As shown in Fig. 2C and in Supp. Fig. 2A, we identified 249 differentially expressed genes (DEGs) that exhibit differential expression upon OD, comparing hBMSC with osteoblasts. The expression of 58 genes was independent of RNF4 (43 DEGs upregulated and 15 DEGs repressed), while107 DEGs exhibited RNF4-dependent increased expression upon OD, and 84 DEGs failed to be repressed upon differentiation in the absence of RNF4 (Supp. Fig. 2B, C). Gene ontology (GO) analysis of OD highlighted the increased expression of genes associated with ossification and cell-death inhibition. It also identified an increase in DEGs linked to innate immunity. Interestingly, RNF4 was required for the upregulation of genes linked to osteoclast differentiation, EGF signaling, the response to LPS, and interferon signaling, possibly representing Toll-related pathways and differentiation (Supp. Fig. 2B, C).

While RNF4 is present in low levels in hBMSCs, it is nevertheless required for OD, suggesting a role in these cells. We, therefore, determined the RNF4-regulated gene signature in hBMSCs by comparing the expression signature of control hBMSCs with that of shRNF4#1-targeted hBMSCs. In undifferentiated hBMSCs, we identified 1172 DEGs whose expression required RNF4, and 790 genes that exhibited ectopic expression upon RNF4 knockdown (Fig. 2D). GO analysis of downregulated genes in hBMSCSCs suggests that RNF4 was required in undifferentiated hBMSCs for the expression of genes that regulate cell morphogenesis and differentiation priming for potential OD. Detailed DEG identity, GO analysis and RNA-seq data are shown in Supp. Fig. 2 and Supplemental Tables S1–S3.

### RNF4-dependent OD requires BMP6 together with its co-receptor RGMb

Since conditioned media from hBMSCs enabled differentiation of RNF4-deficient stalled hBMSCs, we searched our RNA-Seq datasets for factors (soluble or partially anchored to the extracellular cell membrane) whose expression requires RNF4 and that are relevant for OD. We identified the BMP6 ligand and its co-receptor repulsive guidance molecule B (termed RGMb, Dragon) as genes that require RNF4 for their mRNA expression (Fig. 2C–F and Supp. Table S1). BMP6 is well known to promote OD [21], and its mRNA level increased upon differentiation, but not in RNF4-deficient hBMSCs (Fig. 2C and Supp. Table S1). RGMb is a BMP6 co-receptor known to regulate diverse processes such as neuronal guidance and T-cell reactivity, and iron metabolism [34, 40, 41]. Together with BMP receptors such as Neogenin, RGMb allows cells to selectively respond to low levels of BMP ligands [42, 43]. We observed that RGMb is expressed in hBMSCs and its levels remain unchanged during differentiation. Upon RNF4 knockdown, however, RGMb expression in hBMSCs is dramatically reduced (Fig. 2D and Supp. Table S1). Indeed, we validated these RNA-seq results via qPCR and established that both BMP6 and RGMb require RNF4 for their expression (Fig. 2E, F).

**Fig. 3:**
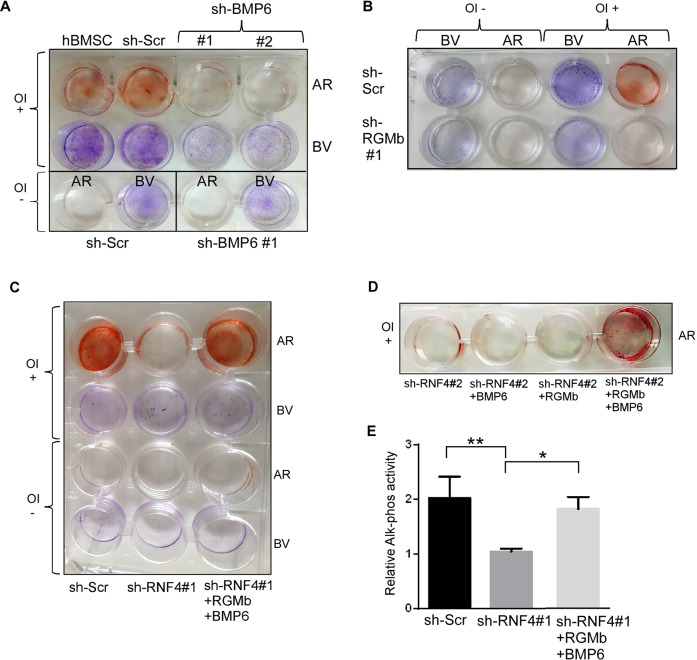
RNF4 promotes hBMSC differentiation via BMP6 and RGMb. **A**, **B** OD for 21 days of the indicated hBMSCs deficient in either BMP6 [shBMP6 (**A**) or RGMb (shRGMb) (**B**). OD analyzed by Alizarin Red (AR) staining. Cell viability was visualized using Brilliant violet (BV) and osteogenic induction is marked by “OI+”. **C**, **D** OD of hBMSCs in which RNF4 was knocked down using the indicated shRNF4. Where indicated, the differentiation media was supplemented with recombinant purified BMP6 or RGMb, or both BMP6 and RGMb proteins as indicated. **E** Alkaline phosphatase activity (Alk-Phos) in the indicated cells 21 days after OI. *n* = 3; **P < 0. 01 *P < 0.1 Statistics was calculated by one-step ANOVA Graph-prism 6.

We hypothesized that BMP6 and RGMb are critical downstream effectors of RNF4, promoting OD. To test this notion, we inactivated each of the factors using multiple independent shRNAs. shRNA-dependent knockdown of either BMP6 or RGMb inhibited hBMSC OD as determined by AR staining and ALP activity (Fig. 3A, B and Supp. Fig. 3C, D).

Moreover, in a set of gain-of-function experiments, the addition of either BMP6 or RGMb as purified recombinant proteins to the culture media was not sufficient to promote differentiation of RNF4-targeted hBMSCs (Fig. 3D). Remarkably, only the co-addition of both proteins to the culture media resulted in robust OD of RNF4-deficient stem cells as evidenced by positive AR staining and high ALP activity (Fig. 3C–E). Taken together, our results suggest that RNF4 promotes OD by enhancing the expression of BMP6 and its co-receptor RGMb, which act together locally to promote OD.

### RNF4, BMP6, and RGMb are essential genes in human cancer

**Fig. 4:**
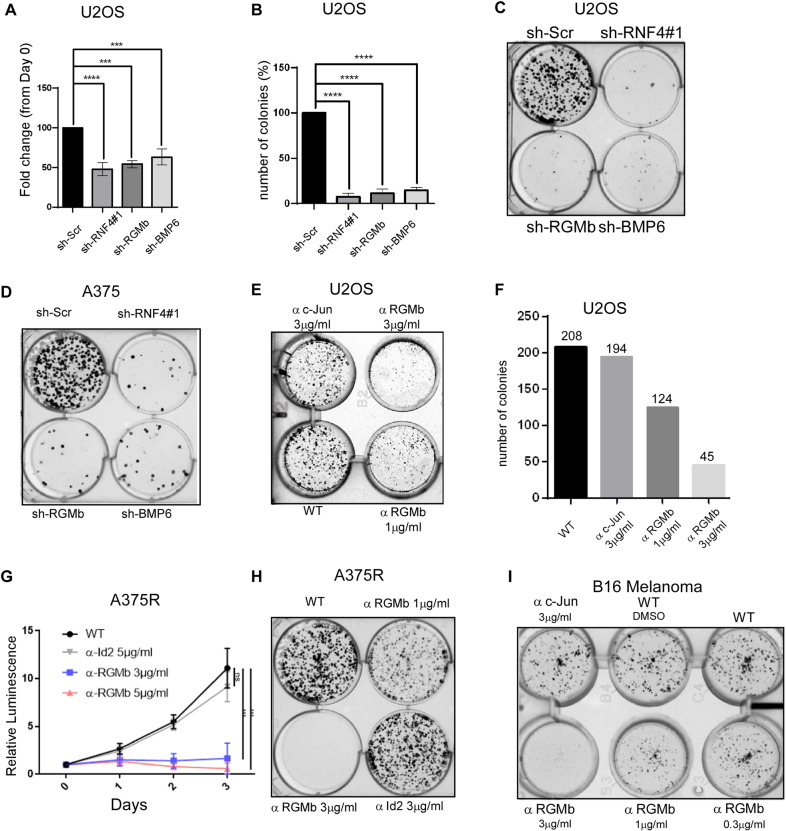
RNF4, BMP6, and RGMb are essential for cancer cell survival and tumor-sphare formation. **A**–**C** shRNA-mediated knockdown of the indicated genes, but not scrambled control (sh-Scr), inhibits cell proliferation (**A**) and sphere formation (**B**, **C**) of human osteosarcoma U-2-OS cells. Cell proliferation was measured indirectly by ATP-Lite assay and in both **A** and **B** *n* = 3 and ****p < 0.001 (**C**) is a representative experiment of (**B**). **D** shRNA-mediated knockdown of the indicated genes, but not scrambled control (sh-Scr), inhibited sphere formation of human A375 melanoma cells. Quantification of the experiment shown in Supplemental Fig. 4B. **E**, **F** Sphere formation of human U-2-OS osteosarcoma cells is inhibited by the addition of α-RGMb, but not by the addition of control antibody (α-c-Jun) to the culture media, in a dose-dependent manner. Quantification of the experiment is shown in **F**. **G**, **H** Proliferation (**G**) and sphere formation (**H**) of human, PLX-4032-resistant melanoma cells (A375R), are inhibited by the addition of α-RGMb, but not by the addition of control antibody (α-Id2) to the culture media, in a dose-dependent manner, *n* = 3 ***p < 0.001. **I** Sphere formation of mouse B16F10 melanoma cells is inhibited by the addition of α-RGMb, but not by the addition of control antibody (α-c-Jun) to the culture media in a dose-dependent manner. Statistics was calculated by one-step ANOVA Graph-prism 6.

Regulators of stem cell potency and differentiation also play significant roles in cancer development [44]. BMP ligands, TGFβ receptors, and SMAD transcription factors play important roles in stem cell differentiation [45]. However and in cancer, these regulators enhance cancer stem cells tumorigenesis, induce epithelial-mesenchymal transition (EMT) and promote metastasis, including in melanoma [45, 46] Likewise, RNF4 is required for *Drosophila* and mouse development, yet it has multiple roles in cancer. For example, RNF4 has tumor suppressive activity in acute promyelocytic leukemia (APL). In APL RNF4 mediates the SUMO-dependent ubiquitination and subsequent degradation of the oncogenic driver PML-RARα, leading to differentiation of the tumor cells [30, 31, 47]. In epithelial cancers and in melanoma, however, RNF4 has a pro-tumorigenic activity [29, 32]. We, therefore, investigated what roles RNF4, BMP6, and RGMb play in osteosarcoma and melanoma cancer cells. shRNA-dependent knockdown of RNF4 reduced RGMb mRNA levels and shRNA-mediated elimination of RNF4, or BMP6, or RGMb attenuated human osteosarcoma U-2-OS cell proliferation, and reduced their ability to form tumor spheres in culture as well as A375 melanoma cells (Fig. 4A–D and Supp. Fig. 4A, B). Since RGMb is present on the surface of hBMSCs and BMP6 is a secreted factor, we used naturalizing antibodies to each of these factors and tested for inhibition of proliferation and formation of tumor spheres of osteosarcoma U-2-OS cells and melanoma A375R cells. The addition of α−BMP6 or control (α−Id2) antibodies to the culture media did not affect cell proliferation or inhibit sphere formation. The addition of α-RGMb antibody, however, reduced proliferation and inhibited sphere formation of human osteosarcoma U-2-OS, as well as PLX-4032-resistant human melanoma cells (A375R), as well as B16 mouse melanoma cells (Fig. 4E–I and Supp. Fig. 4C–E).

**Fig. 5:**
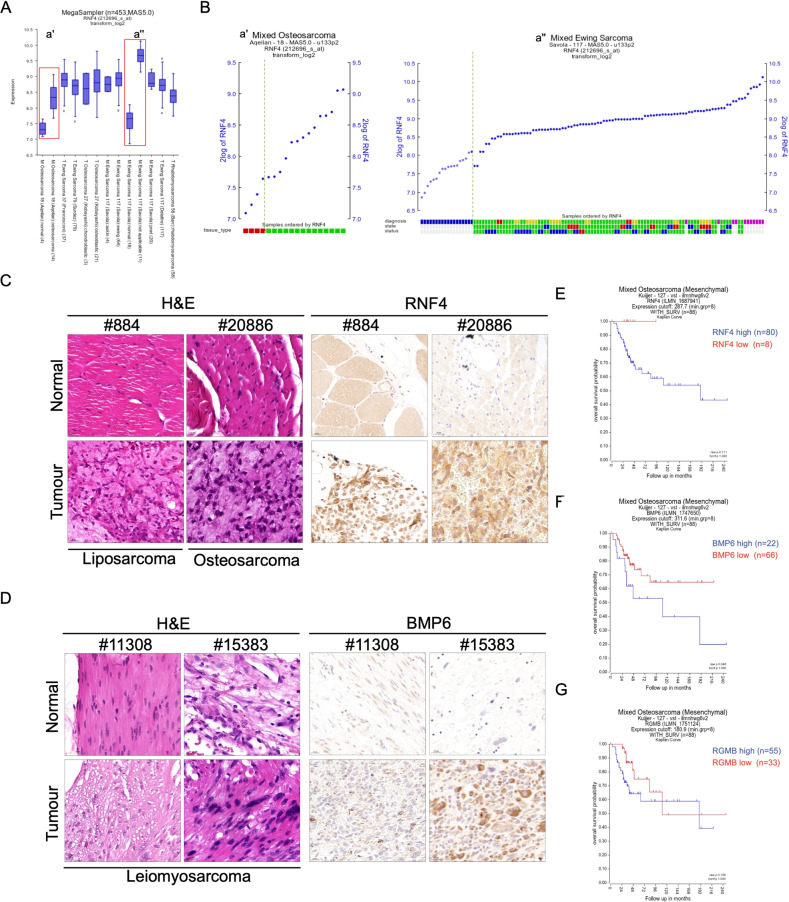
RNF4 and BMP6 levels are upregulated in sarcomas and are prognostic markers for poor survival. **A** Box-plot of RNF4 mRNA expression derived from publicly available human sarcoma samples. Sample size, “*n*”, is indicated in brackets. For the mixed osteosarcoma expression set by Aqelian, as well as the Mixed Ewing Sarcoma dataset by Savola (highlighted in red boxes), RNF4 expression in normal tissue and sarcoma samples are presented individually in (**B**). Data generated with the online R2 visualization tool, Genomics Analysis, and Visualization Platform (https://hgserver1.amc.nl/cgi-bin/r2/main.cgi). **B** Individual RNF4 mRNA expression levels of samples within the mixed osteosarcoma expression set by Aqelian (Left panel; red = normal, green = tumor) and the Mixed Ewing Sarcoma dataset by Savola (right panel; diagnosis: blue = normal, green = Ewing, red = askin, yellow = pnet, purple = non-classified; state: green = primary, red = metastasis, blue = recurrent; status: green = diseased, red = no evidence of disease (ned), red = alive with disease (awd)). Data were generated with the online R2 visualization tool, Genomics Analysis and Visualization Platform (https://hgserver1.amc.nl/cgi-bin/r2/main.cgi) and adapted to present normal vs. tumor samples. The green dotted line separates the normal and tumor samples. **C**, **D** Representative immuno-staining using antibodies against RNF4 and BMP6 on human pan-sarcoma TMA (*n* = 30). In each set, normal non-tumor tissue is shown. **C** Hematoxylin and eosin (H&E) and α-RNF4 staining of representative cases of human pan-sarcoma tissue microarray comprising 30 cases; Liposarcoma #884; Osteosarcoma #20886. **D** H&E and α-BMP6 staining of representative cases of human pan-sarcoma tissue microarray comprising 30 cases. In each set, normal non-tumor tissue is shown. Samples #11308 and #15383 are both leiomyosarcoma. **E**–**G** Kaplan–Meier plots of mixed osteosarcoma overall patient survival relative to RNF4, BMP6, and RGMB expression. Blue = high expression, Red = low expression. Total sample size *n* = 88. Data generated with the online R2 visualization tool, Genomics Analysis and Visualization Platform (https://hgserver1.amc.nl/cgi-bin/r2/main.cgi). To establish the optimum survival cut-off by statistical testing, classification in high and low expression was performed using the R2 Kaplan Scan function. The expression graph illustrates *p* value plotted against mRNA expression to determine the best cut-off. Statistical significance, *P*, was calculated by log-rank test.

To evaluate the clinical relevance of our experimental findings, we compared the mRNA expression level of RNF4 in various kinds of human sarcomas. As shown in Fig. 5A, B, higher levels of RNF4 mRNA were observed in osteosarcoma and Ewing sarcoma tumors compared with the adjacent, normal tissue (Fig. 5A red squares, 5B). Moreover, RNF4 levels were high also in other sarcomas (Fig. 5A). Analysis of patient samples for mRNA co-expression revealed statistically significant co-mRNA expression of either BMP6 and RGMb with RNF4 (Supp. Fig. 5A). RNF4, BMP6, and RGMb are not, however, mutated in sarcoma patients (Supp. Fig. 5B). We, therefore, tested patient-derived sarcoma tissue microarray (TMA) for the protein levels of RNF4 and BMP6 and RGMb. While technically, we were unsuccessful in performing immunohistochemistry of RGMb in these biopsies, these tumors were characterized by high levels of RNF4 and BMP6 compared with non-tumor tissue, as well as higher levels of the proliferation marker Ki67 (not shown). For example, Fig. 5C, D, and Supp. Fig. 5C–F shows representative cases of liposarcoma (#884), osteosarcoma (#l20886), and leiomyosarcoma (#11308, #315383). Overall, in all tumor portions of the tissue microarray (TMA) we observed higher levels of RNF4 and BMP6 compared to the non-tumor tissue. These observations are clinically important since we found that both overall and disease-free survival of sarcoma patients exhibiting high levels of RNF4 or BMP6 (but not RGMb) was shorter than that of patients exhibiting low RNF4 or BMP6 levels (Fig. 5E–G) Moreover, high RNF4 levels were associated with reduced overall survival, progress free survival, and disease-free survival (Supp. Fig. 5G). Taken together, our data suggest that RNF4 and its downstream target genes, BMP6 and RGMb, are essential for the survival of osteosarcoma and RTKi-resistant melanoma cells and that RNF4 and BMP6 may serve as markers associated with poorer prognosis in sarcomas.

## Discussion

Our study describes the role of the RNF4~RGMb~BMP6 axis in the osteogenic differentiation (OD) of hBMSCs. We also discovered that these factors are essential for the survival and tumorigenicity of osteosarcoma and melanoma cancer cells. RNF4 and BMP6 mRNA and protein levels are highly elevated in multiple types of human sarcomas and are associated with poor prognosis and reduced patient survival.

### RNF4 and differentiation

RNF4 and its orthologs are required for embryonic development and differentiation [48**–**53]. In *Drosophila melanogaster* embryogenesis, the RNF4 ortholog Degringolade (Dgrn, CG10981) regulates segmentation, sex determination, and neurogenesis [48, 49]. In these processes, Dgrn acts in part by inactivating the co-repressor Groucho (Gro/TLE) [49].^.^In Zebrafish (Danio rerio), RNF4 is required for granulopoiesis, and the zebrafish *rnf4* knockout line exhibited a dramatic reduction in neutrophil numbers. This was likely due to the continuous repression of the CCAAT/enhancer-binding protein α (C/EBPα) transcription factor via promoter hypermethylation by the SUMOylated DNA methyltransferase 1 (DNMT1) [53]. Likewise, RNF4 protein accumulated during the late stages of spermatogenesis, suggesting a role in the terminal differentiation of germ cells [54].

Remarkably, RNF4 promotes the arsenic-induced differentiation of leukemic cells into granulocytes by targeting the SUMOylated PML-RARα oncoprotein for degradation. As a result, arsenic therapy is a standard treatment for APL [47].

Dgrn and RNF4 were shown in the above studies to enable SUMO-dependent transcriptional de-repression. Moreover, STUbL proteins were shown to enhance chromatin accessibility [55, 56]. RNF4 is known to interact with nucleosomes via its nucleosome-targeting motif, an interaction that is required for its transcriptional activity. [28, 29]. Both RNF4 and Dgrn proteins were shown to actively enhance gene expression. RNF4 is a co-activator of the androgen receptor, and both Dgrn and RNF4 enhance transcriptional activation of the Notch, Wnt, and NF-κB pathways [29, 48, 51, 57]. Gene activation by RNF4 and its orthologs involves several mechanisms, including global de-methylation [55]. We found that RNF4 is required during OD for the expression of BMP6 and its co-receptor RGMb, which acts locally on the differentiating progenitors.

The exact transcriptional mechanisms by which RNF4 enhances the expression of these factors, is currently the focus of an ongoing study.

### RNF4-regulated gene signature in hBMSCs and OD

Genomic analyses of RNF4-regulated gene expression signatures identified a specific yet broad RNF4-regulated gene signature in hBMSCs that is distinct from the RNF4-regulated genes during OD with only minimal overlap. This set of RNF4-regulated genes is also distinct from RNF4-regulated genes in melanoma tumors [32].

Interestingly, SUMO-peptidase 3 (SENP3) was required for the OD of human dental stem cells [58]. Nayak et al. discovered that SENP3 is required for the de-SUMOylation of RbBP5, which is part of the histone methyltransferase MLL1/2 COMPASS complexes. This activity of SENP3 is required for the transcriptional activation of distinct HOX transcription factors such as DLX3, which is essential for OD. DLX3 or SENP3 are not, however, regulated transcriptionally by RNF4 in hBMSCs, and unlike in the case of SENP3, DLX3 mRNA level is not reduced in the absence of RNF4 upon OD. This suggests that RNF4 and SENP3 likely regulate parallel pathways of OD.

RNF4 was previously identified as a gene that is shared by neuronal, hematopoietic, and pluripotent stem cells [33]. The large set of genes regulated by RNF4 in hBMSCs is related to maintaining progenitor identity as well as their ability to differentiate. Analysis of RNF4-regulated genes also suggested that RNF4 is a pro-survival regulator of progenitors, as it represses the expression of P53-related cell-death pathways. The transcriptional signature of RNF4 in hBMSCs fits well with the observations that progenitors are stalled in the absence of RNF4 and complements our factor supplementation experiments. It is not clear, however, to what extent is this gene signature a general progenitor RNF4-regulated gene signature and which part of this signature is hBMSC- and OD-specific.

### BMP6, RGMb, and OD

Two factors that are tightly linked to OD are BMP6 and RGMb, which were identified as target genes of RNF4. BMP6 is a potent TGF-β ligand family member and is a well-known driver of osteogenesis and chondrogenesis [43]. BMP6 interacts with its extracellular co-receptor RGMb to enhance the cellular response to BMP6. RGMb is a glycol-phosphatidyl inositol-anchored membrane protein that associates with canonical receptors such as type-II BMP receptors, Activin receptors, and Neogenin (Neo1), fostering BMP signaling [59**–**62]. For example, RGMb is required for BMP signaling during endochondral bone formation [43]. Likewise, BMP6~Neo1~RGMb trimeric complex is essential for the differentiation of the olfactory epithelium, where it regulates interactions between stem cells and newly born neurons [42]. Moreover, the RGMb complex regulates the activity of the immune system in the lung epithelia via binding to PD-L2 [63, 64]. Our results suggest that RGMb is already expressed in undifferentiated hBMSCs and that its expression in hBMSCs requires RNF4, likely priming the responsiveness to BMP6. The observation that only the co-addition of both factors to the culture media restores OD suggests a molecular interaction between BMP6 and RGMb on the cell surface. However, the distinct receptor(s) that act together with BMP6 and RGMb, as well as the exact downstream signaling pathway in the context of OD and require further exploration.

### RNF4, RGMb, and BMP6 in cancer

RNF4 has multiple faces in cancer. Here, we report that RNF4 via BMP6 and RGMb is required for tumor cell survival and tumorigenicity of osteosarcoma and RTK inhibitor-resistant melanoma cells (A375R). Likewise, RGMb is a new player in cancer biology and has both anti- and pro-tumorigenic activities that are cancer-type-dependent. While in breast cancer, RGMb and BMP signaling have a suppressive function, in colon cancer, RGMb has pro-oncogenic activities [65, 66]. Moreover, our transcriptional analyses suggest that BMP6 and RGMb are situated downstream of RNF4. Thus, the expression of RNF4 modifies the environment in the vicinity of cancer cells, promoting tumor cell survival. Part of RNF4 enhancing tumorigenic activity involves the stabilization of oncoproteins as well as conferring resistance to RTK inhibitor therapy [32]. However, our preliminary unpublished results do not support a significant role for RGMb or BMP6 in oncoprotein stabilization. Thus, suggesting that the pro-tumorigenic activity of RNF4 is multifaceted and that the RNF4-RGMb-BMP6 pathway acts primarily as a pro-survival pathway in sarcomas and therapy-resistant cells, and that BMP6 and RGMb inhibition has a potent anti-cancer activity. However, future studies are required to fully elucidate the RNF4-dependent molecular axis in therapy-resistant tumors and to elucidate the full tumor spectrum where this axis acts as a pro-survival pathway.

### Clinical relevance of our observations to bone regeneration and sarcomas

The observation that BMP6 and RGMb are positioned downstream to RNF4 and can together alleviate the stall in progenitor differentiation may have the potential for bone regeneration therapy. Thus, a future experiment should explore whether supplementing BMP6 and RGMb to regenerating hBMCs may be a powerful method for potentiation of bone regeneration and differentiation. However, given the role of BMP6 and RGMb as survival factors in sarcoma, these experiments should also address a potential transformation/tumor-promoting aspect of these factors, as they may be a “double adage sord”.

Remarkably, and in cancer, high levels of RNF4 and BMP6 were associated with poor prognosis and shorter disease-free survival in multiple sarcoma types. RGMb showed a positive correlation for expression with RNF4 in patient sample. However, unlike RNF4 and BMP6, high levels of RGMb do not correlate with poorer prognosis and we currently do not fully understand this difference.

Taken together, our study highlights the important roles of RNF4 and its regulated factors RGMb and BMP6 in osteogenic differentiation of mesenchymal stem cell and cancer opening the door for the development of diagnostics and specific inhibitors for the treatment of these challenging cancers.

## Material and methods

### Antibodies

αRNF4 8D10mAb antibody and rabbit polyclonal αRNF4 were used as described [29]. α− β-catenin (1:1000, #9564) ATF-4 (D4B8) Rabbit mAb (1:1000, #11815) were obtained from Cell Signaling Technology. Mouse α-Actin (1:1000) was from MP Biomedicals. α-CD90 (#328109); α-CD45 (#304014) and α-CD31 (#3030103) were all from Biolegend, and α-CD44 (#17-0441-82) was from EBioscience. α−RGMb was from Lsbio (LS-C185861). α−Neo1 was from xxx, and α- c-Jun (H-79) was from Santa Cruz Biotechnology.

#### Primers used for qPCR

hRNF4 fwd: 5′-CCCGAGATCTCCTTGGAAGC-3′

hRNF4 rev: 5′-TCATCGTCACTGCTCACCAC-3′

hBMP6 fwd: 5′- CAGCCTGCAGGAAGCATGAG -3′

hBMP6 rev: 5′- CAAAGTAAAGAACCGAGATG -3′

hRGMB fwd: 5′- GGCCTGGCCACTCATAGATA -3′

hRGMB rev: 5′- GCGGCAGTAAAGTTGGCATCAC -3′

hRunx2 fwd: 5′- ATGGGACTGTGGTTACTGTCATGGCGGG -3′

hRunx2 rev: 5′- CTGGGTTCCCGAGGTCCATCTACTGTAACTTTAATTGC -3′

GAPDH fwd: 5′-ACATCAAGAAGGTGGTGAAGCAGG-3′

GAPDH rev: 5′-AGCTTGACAAAGTGGTCGTTGAGG-3′

#### Plasmids and viral expression constructs

Plasmids coding for HA-RNF4 and pLKO-based shRNF4, scrambled (sc-shRNA; control), were all as described in ref. [29].

shRNF4 #1:

5׳CCGGGACAAGCTCAGAAGCGAACTCCTCGAGGAGTTCGCTTCTGAGCTTGTCTTTTTG-3׳.

shRNF4 #2:

5′-CCGGCATCTGCATGGACGGATACTCCTCGAGGAGTATCCGTCCATGCAGATGTTTTTG-3′

shBMP6: 5′- CCGGCGCACACATGAATGCAACCAACTCGAGTTGGTTGCATTCATGTGTGCGTTTTTG-3′

shRGMB: 5′- CCGGACTCACCTGCTTGATCCTTATCTCGAGATAAGGATCAAGCAGGTGAGTTTTTTG-3′

shScrambled: 5′-CCGGGCCCCAACTCGATAGAGAAGACTCGAGTCTTCTCTATCGAGTTGGGGCTTTTT-3′

#### Cultured cell lines and media

U-2-OS, A375, HEK293T, and B16F10 melanoma cell lines were from ATCC. A375R cell line was described elsewhere [32]. Cells were maintained in DMEM with 100 U/ml penicillin, 0.1 mg/ml streptomycin, and 10% fetal bovine serum (FBS).

B16F10 were maintained on RPMI with glutamine 2 mM, neomycin 2 mM, sodium pyruvate solution 2 mM, MEM non-essential amino acids solution (100X) 2 mM, MEM vitamin solution (100X) 3 mM, sodium bicarbonate solution (7.5%) 15 mM, and 10% FBS. hBMSCs were maintained in αMEM medium with 100 U/ml penicillin, 0.1 mg/ml streptomycin, and 10% FBS.

##### hBMSC isolation and maintenance

hBMSCs were isolated from bone marrow aspirates based on their adhesion to plastic culture dishes and were cultured in minimum essential medium alpha (MEMα) supplemented with 10% FBS, 2 mM l-glutamine, and Pen-Strep (both 100 U/ml). The medium was replaced every 3 days until the hematopoietic cells were washed away, leaving a homogenous adherent culture of hBMSCs. hBMSCs were further expanded and passaged for no longer than three passages

##### Osteogenic induction

To induce osteogenesis, hBMSCs were cultured under osteogenic induction conditions (culture medium supplemented with 100 μg/ml ascorbic acid-magnesium, 10–8 μM dexamethasone, 10 mM sodium β-glycerophosphate). During osteogenesis, the osteogenic medium was replaced every 3 days. All media components were from Biological Industries, Beit HaEmek, Israel.

#### Osteogenic induction using medium transfer/recombinant BMP6 and RGMB proteins

hBMSCs were cultured under osteogenic induction conditions as described above. Every 3 days during the osteogenesis, the osteogenic medium was harvested from non-differentiating or differentiating hBMSCs. The collected medium was filtered through a Millipore 0.45-micron syringe filter and added to the media of hBMSC cells where RNF4 was knockdown. The procedure was repeated every 3 days throughout the experiment. Where indicated, human recombinant proteins were added to the osteogenic medium according to the experimental design for final concentrations of 100 ng/ml for RGMb, and 60 ng/ml for BMP6. The osteogenic medium with supplemental recombinant proteins was replaced every 3 days.

##### Transfections and infections

For virus production, cells were transfected with lentiviral packaging vectors MD2G and PPAX using CalFectin transfection reagent (Sinagen Laboratories) according to the manufacturer’s instructions.

##### RNA-sequencing and data analyses

RNA-seq data analysis was performed at the Genomics Center at the Biomedical Core Facility, Technion. We performed RNAseq on four experimental groups with three biological replicates for each group: Group 1. Control hBMSCs following infection with scrambled shRNA, prior to osteogenesis induction (day zero). Group 2. Control hBMSCs following infection with scrambled shRNA, 7 days after osteogenesis induction. Group 3. hBMSCs where RNF4 was knockdown using shRNF4 at day zero. Group 4. hBMSCs where RNF4 was knockdown 7 days after osteogenesis induction. RNA extraction and QC quality control for total RNA were performed using TapeStation (Agilent). The RINe value of all samples was in the range of 8.0–9.8, indicating that all samples passed the QC analysis. Raw data was generated using 12 RNA-seq libraries (NEBNext UltraII Directional RNA Library Prep Kit for Illumina, cat. no. E7760) were produced according to the manufacturer’s protocol using 800 ng total RNA. mRNA pull-up was performed using a Magnetic Isolation Module (NEB, cat. no. E7490). All 12 libraries were mixed into a single tube with equal molarity. The RNA-seq data was generated on Illumina NextSeq500, 75 cycles, high-output mode (Illumina, cat. no. FC-404-2005). NGS QC, alignment, and counting quality control was assessed using Fastqc (v0.11.5), reads were trimmed for adapters, low quality 3′, and a minimum length of 20 using CUTADAPT (v1.12). 80 bp single-end reads were aligned to human reference genome (Homo_sapiens.GRCh38.dna.primary_assembly.fa downloaded from ENSEMBL) and annotation file (Homo_sapiens.GRCh38.92.gtf downloaded from ENSEMBL) using STAR aligner (v2.6.0a). The number of reads per gene was determined using Htseq (v0.9.1).

##### Bioinformatics analysis

Statistical analysis was performed using the DESeq2 R package (v1.18.1) [67]. The number of reads per gene was extracted into Count.xls and NormalizedCounts.xls files for raw counts and normalized counts, respectively. The similarity between samples was evaluated in the DESeq2 package using a correlation matrix. A list of differentially expressed genes in all experiments (DEGs) are available in an excel file (DESeq2_results.xls).

##### Interactions, pathways, and networks analysis

GO analysis was generated using Cytoscape software (v3.9.1) [ClueGO app (v2.5.8) for GO enrichment analyses[68, 69].

##### RT-PCR

RNA was extracted from cell pellets using RNAmicro Isolation Kit (QIAGEN Cat:). cDNA was synthesized from 1 µg of RNA using qScriptTM cDNA Synthesis Kit (Quanta Biosciences). The PCR reaction was performed with StepOnePlusTM Real-Time PCR System (Thermo Fisher) using SYBR Green (Quanta Biosciences) for detection. Amplification was carried out in a 25 µL reaction volume for 45 cycles of 15 s at 95 °C and 60 s at 60 °C. The housekeeping gene GAPDH was used as an internal control for normalization.

##### Alizarin red staining

Cells were washed twice with PBS, fixed for 20 min with 4% paraformaldehyde at room temperature, washed three times with PBS, and finally stained with Alizarin red solution (Sigma-Aldrich) for 5 min at RT. The excess staining solution was washed away with water and cells were then dried out. For comparative evaluation of the results, duplicates of the experimental samples were stained with 0.05% crystal violet for 20 min at RT.

##### Alkaline phosphatase activity

Lysates for alkaline phosphatase activity were obtained by adding 0.25 mL cold lysis buffer (1 mM MgCl2, 0.5% Triton-X100 in Alkaline Buffer Solution (Sigma)) per sample. After incubating for 1 h on ice, another 0.25 mL of cold lysis buffer was added. For the reaction, 100 µL lysate and 400 µL phosphatase substrate solution (20 mg p-nitrophenol phosphate (Sigma-Aldrich) in 1.25 mL alkaline buffer solution and 3.75 mL ddH2O) were incubated in a water bath at 37 °C for 10 min. The reaction was stopped by adding a 500 µL stop solution (20 g NaOH, 37.22 g Na2EDTA in 500 mL ddH2O). Absorbance was read at 405 nm using the ELISA Zenith 200 (Anthos Labtec). ALP activity was calculated using serial dilutions of 4-nitrophenol solution (10 mM).

##### Proliferation and sphere formation assays

Cancer cell survival and sphere formation assays were performed as described elsewhere [32]. In brief, cell viability was determined using MTT solution (Abcam #146345) and ATPlite assay kit (CellTiter-Glo Promega #G7570) according to the manufacturer’s instructions. In assays evaluating resistance to PLX-4032, PLX-4032-treated cells were cultured for 5 days before analysis. For the ATPlite assay, viability was quantified by monitoring luminescence intensity according to the manufacturer’s instructions. Viability was quantified by colorimetry using Stat Fax 2100 ELISA reader. For sphere formation assay, U-2-OS and melanoma cells (2000 cells/well) were seeded in six-well plates in 2–3 ml DMEM or RPMI media, respectively, and were maintained at 37 °C in a humidified incubator. After 6–8 days, cells were fixed overnight with 5% formaldehyde, washed thrice with phosphate-buffered saline (PBSx1), stained with 0.05% crystal violet for 20 min, photographed, and counted.

##### Histopathology and human sarcoma TMA

For IHC and H&E, slides were de-paraffinized and rehydrated following the previously reported protocol [70]. In brief, IHC slides were subjected to epitope retrieval and blocked in 3% BSA at RT for 1 h. The antibody manufacturer’s instructions were followed for all antibodies. In general, primary antibodies (diluted in 1% BSA) were incubated overnight at 4 °C, followed by three washes with PBS and subsequent incubation with the DAB secondary antibody for 1 h at RT. Then, slides were washed twice with 1xPBS for 5 min and stained with the DAB staining solution in 1xPBS. Upon DAB staining, slides were counteracted with hematoxylin and washed thrice with 1xPBS for 5 min. Slides were mounted using 200 μl of Mowiol® 40–88 covered by a glass coverslip. IHC slides were recorded using a Panoramic DESK scanner or FSX100 microscopy system (Olympus) and analyzed using Case Viewer software (3DHISTECH), QuPath software, and ImageJ. The antibodies used were: anti-RNF4 (Merck, HPA022047) and BMP6 (Merck, HPA062683).

#### Patient-derived tissue microarrays (TMA)

Paraffin molds were cast using an Arraymold Kit (IHC World, Kit D, IW-115, core diameter 2 mm, 36 cores). Human samples were cut and stained using hematoxylin and eosin and digitalized using a 3D Histech slide scanner (panoramic FLASH). Tumor and non-transformed tissue were identified and manually ‘punched’ and transferred from the tissue block to the tissue array. Upon completion, 3-µm thick sections were cut using a microtome and processed as described above.

##### Analysis of publicly available data

All publicly available data and software used for this publication are listed (Appendix Table S5). Oncoprotein mutational plots and patient survival were generated using cBioportal [71, 72]. In brief, cBioportal generates graphical representations of genomic alterations, somatic mutations, copy number alterations, and mRNA expression changes. Correlation analyses were performed using the GEPIA2 online tool (http://gepia2.cancer-pku.cn/#index). The analysis was based on the expression of RNF4 and either RGMB or RNF4 relative to RGMB, BMP2, BMP3, BMP6, NOTCH1, and CTNNB1, factors that are involved in sarcoma proliferation. Data underlying the correlation analysis were “TCGA SARC tumors” and “TCGA SARC normal”. *p* values for Pearson correlation coefficients were calculated using two-tailed Student’s *t*-tests.

For patient expression of sarcoma patients (Fig. 5A, B, E–G), the online R2 tool Genomics Analysis and Visualization Platform (http://r2.amc.nl), was used to visualize gene expression data. *p* values were computed using a log-rank test. For patient survival and Kaplan–Meier blots plots of mixed osteosarcoma, overall patient survival data were generated using the online visualization tool R2: Genomics Analysis and Visualization Platform (https://hgserver1.amc.nl/cgi-bin/r2/main.cgi). To establish the optimum survival cut-off through statistical testing, classification in high and low expression was established using the R2 Kaplan Scan function. The expression graph illustrates the *p* value plotted against mRNA expression to determine the best cut-off. Statistical significance, *P*, was calculated using the log-rank test.

##### Statistical analyses

Statistical analysis, SEM, and *t*-test comparisons were performed using one-step ANOVAs software GraphPad Prism 6 and. In all experiments, significance is as follows: ****p < 0.0001, ***p < 0.001, **p < 0.01, *p < 0.05.

##### Contact for reagent and resource sharing

Further information and requests for resources and reagents should be directed to and will be fulfilled by the Lead Contact, Amir Orian (mdoryan@technion.ac.il).

## Supplementary information

Legend to supplemental figures

Supp figure 1

Supp Figure 2

Supp Figure 4

Supp Figure 5

Table S1

Table S2

Table S3

checklist

## Data Availability

RNA-sequencing data is available at the Gene Expression Omnibus under accession number GEO: Accession ID: GSE205432.
