# Supplementary material for: RNF4~RGMb~BMP6 axis required for osteogenic differentiation and cancer cell survival
Source: Cell Death Dis. 2022 Sep 24;13(9):820. doi: 10.1038/s41419-022-05262-1 (PMC9509360; doi:10.1038/s41419-022-05262-1)
Supplement: Supplementary file 1 — Legend to supplemental figures [file 41419_2022_5262_MOESM1_ESM.docx]

**Supplemental Figure 1: (A,B)** Original full gels western blots used in Figure 1A, B. **(C)** Western blot of U2-OScellsexpressing HA-RNF4 infected with the indicated shRNA. Actin serves as loading control. **(D-F)** FACS-assisted analysis of OD of the indicated cell surface markers at day zero,and fourteen days of OD. Representative FACS analysis is shown, and bar-graph represent three independent biological experiments. CD31 and CD45 are hemotopetic markes that are minimally expressed on hBMCs and do not change upon OD nor do they change upon loss of RNF4. n=3 ***=p<0.001; **=p<0.01. Statistics was calculated by One-step ANOVA Graph-prism 6

**Supplemental Figure 2:**  **Cytoscape-based GO analysis of RNF4-dependent and independent gene signatures during OD** (Complementing Figure 2C). GO analysis of: **(A)** DEGs during OD. **(B)** RNF4-regulated genes during OD. **(C)** GO analysis of RNF4-indpendent genes during OD.

**Supplemental Figure 3: Loss of BMP6 or RGMb inhibits OD. (A, B)** Alkaline phosphatase activity upon OI of control scrambled hBMSCs, or hBMSCs in which BMP6 (A) or RGMb (B) were eliminated using shRNA. **(C, D)** qPCR analysis of BMP6 (A) and RGMb (B) mRNA levels in hBMSCs infected with the indicated shRNA, 21 days after OI in parallel wells to those in Figures 3A, B; (n=3 **** =p<0.0001). Statistics was calculated by One-step ANOVA Graph-prism 6

**Supplemental Figure 4**: RGMb is required for melanoma cell survival. **(A)** mRNA expression level of RNF4 and RGMb in A375R cells treated with scrambled control (sh-Scr) or shRNA against RNF4 (shRNF4#1). (**B)** Proliferation of human A375R PLX-4032-resistant cells is inhibited by the addition of α-RGMb, but not by the addition of control antibody, to the culture media, in a dose-dependent manner, n=3. **= P<0.01.

**(C, D)** sphere formation of human, PLX-4032-resistant cells (A375R), is inhibited by the addition of α-RGMb. (C) is a representative experiment and (D) is quantification of 3 independent biological repeats. **= p<0.01; n.s.= non-significance.

**(E)** Proliferation of human, PLX-4032-resistant cells (A375R), is inhibited by the addition of α-RGMb in a dose dependent manner. Statistics was calculated by One-step ANOVA Graph-prism 6

**Supplementary Figure 5:** **RNF4 in human sarcomas** **(A)** Correlation data of mRNA expression between RNF4 and RGMb, or RNF4 and BMP6 in a publicly available pan-sarcoma data set using the online tool ( http://[gepia2](http://gepia2.cancer-pku.cn/#index).cancer-pku.cn/#index ) R=Pearson correlation coefficient. **(B)** Schematic representation of mutations in RNF4, BMP6, and RGMb in human pan-sarcoma samples; n=251. Data generated with the TCGA online visualization tool (www.cbioportal.org). **(C-F)** H&E staining of tumor and non-tumoral tissue of samples shown in Fig. 5C. **(G)** Kaplan-Meier plots of pan-sarcoma patients’ overall, progression-free, disease-free and disease-specific survival, relative to RNF4 expression for total number of patients. Median survival in months are highlighted for each sub-graph. Total sample size is n=251. Statistical significance, P, was calculated by LogRank test, allowing to test hypothetical survival distribution of two groups. Data were generated with the TCGA online visualization tool www.cbioportal.org.
