# Supplementary figures and images for: RNF4~RGMb~BMP6 axis required for osteogenic differentiation and cancer cell survival
Source: Cell Death Dis. 2022 Sep 24;13(9):820. doi: 10.1038/s41419-022-05262-1 (PMC9509360; doi:10.1038/s41419-022-05262-1)

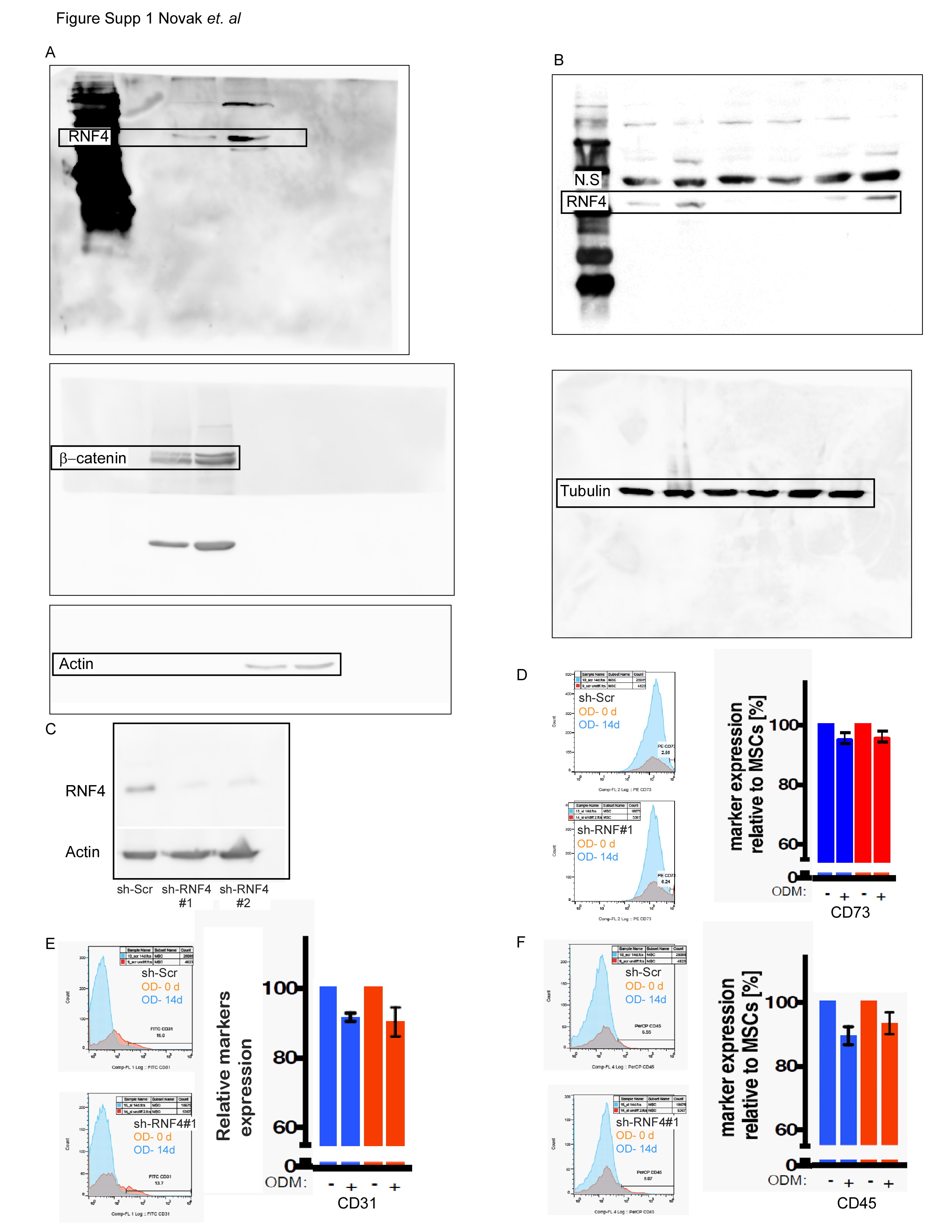

Supplement: Supplementary file 2 — Supp figure 1 [file 41419_2022_5262_MOESM2_ESM.jpg]

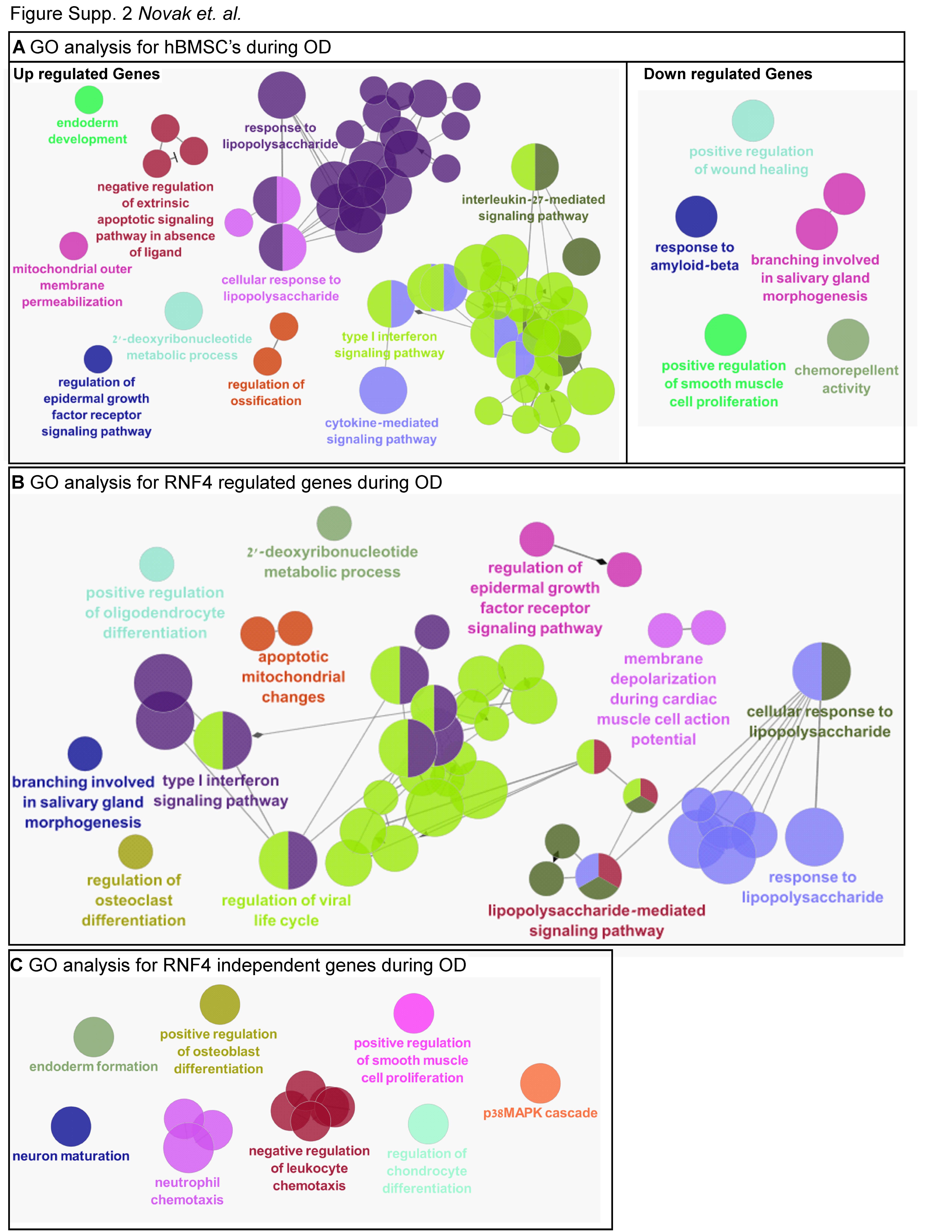

Supplement: Supplementary file 3 — Supp Figure 2 [file 41419_2022_5262_MOESM3_ESM.jpg]

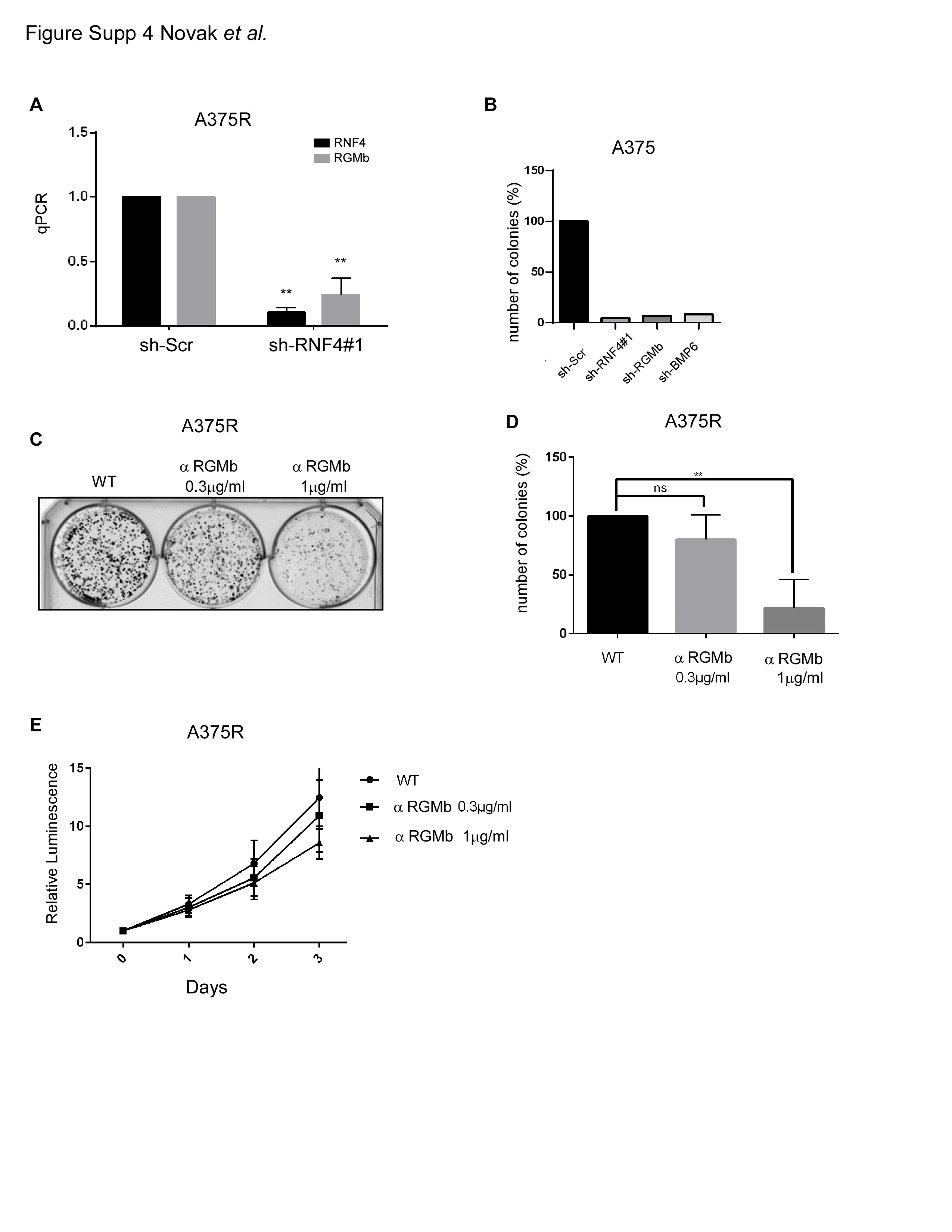

Supplement: Supplementary file 4 — Supp Figure 4 [file 41419_2022_5262_MOESM4_ESM.jpg]

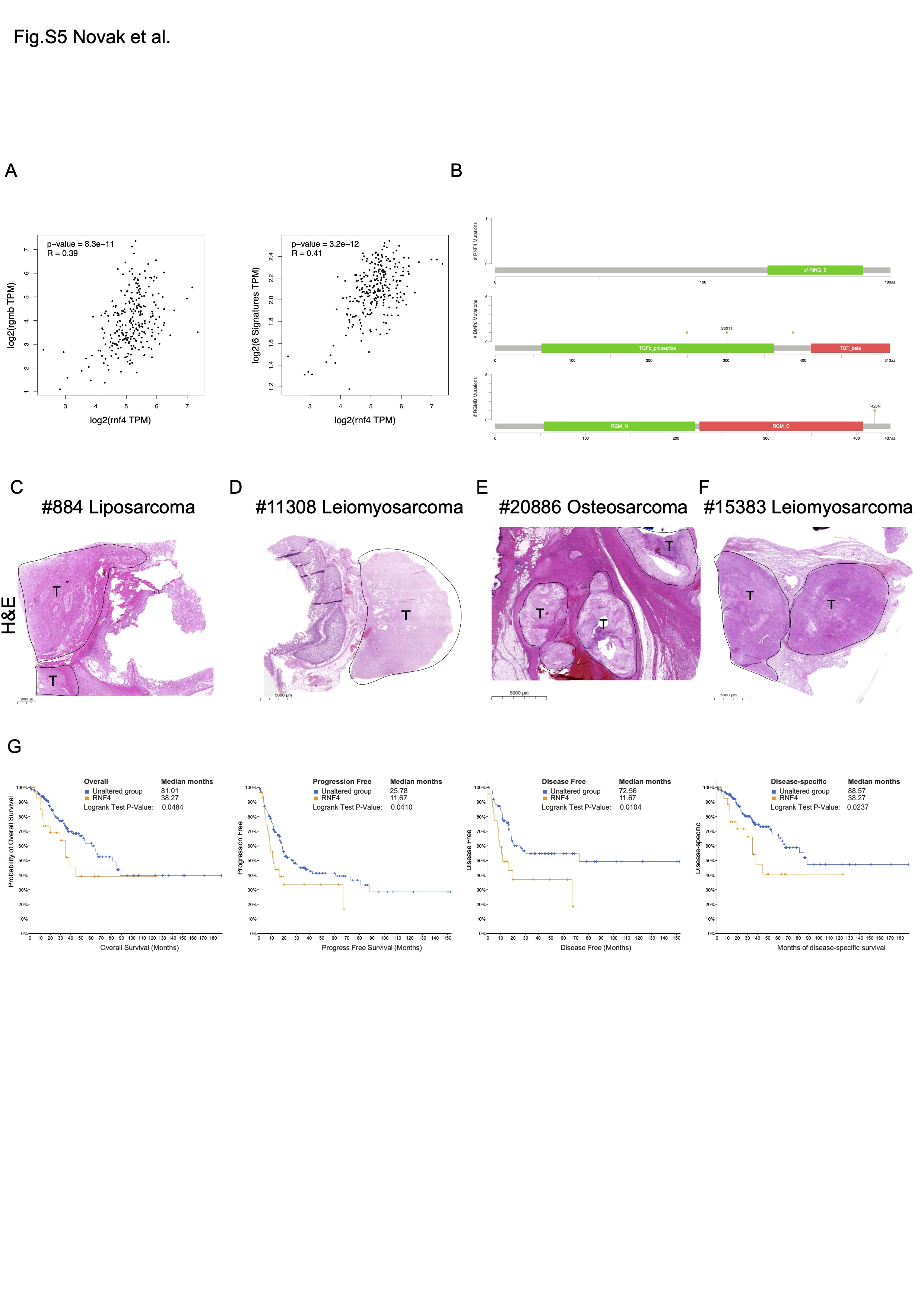

Supplement: Supplementary file 5 — Supp Figure 5 [file 41419_2022_5262_MOESM5_ESM.tif]
